# Chronic diarrhea as a presentation of Behçet's disease

**DOI:** 10.1002/ccr3.7359

**Published:** 2023-05-18

**Authors:** Marawan Elmassry, Sayed Matar, Jerapas Thongpiya, Pitchaporn Yingchoncharoen, Mostafa Abohelwa, Sameer Islam

**Affiliations:** ^1^ Department of Internal Medicine Texas Tech university Health Sciences Centre Lubbock Texas USA; ^2^ Department of Pathology Brigham and Women Hospital, Harvard Medical School Boston Massachusetts USA; ^3^ Department of Gastroenterology and Hepatology Texas Tech university Health Sciences Centre Lubbock Texas USA

**Keywords:** aphthous ulcer, Behcet's disease, diarrhea, DMARDs, inflammatory bowel disease, pathergy

## Abstract

**Key Clinical Messages:**

Behçet's disease (BD) or syndrome is a chronic, recurrent, multisystem, inflammatory vasculitis disorder with findings of oral aphthous ulcers, genital ulcers, and uveitis. Gastrointestinal (GI) involvement can be the initial presentation as presented in this case.

**Abstract:**

Behçet's disease (BD) or syndrome is a chronic, recurrent, multisystem, inflammatory vasculitis disorder of unknown etiology with classical findings of oral aphthous ulcers, genital ulcers, and ocular involvements including chronic anterior, intermediate, posterior, and even panuveitis. Gastrointestinal involvement in BD usually presents with chronic diarrhea, hematochezia as the disease affects ileocecal area which might be similar to presentation of inflammatory bowel diseases. Here, we report a case of undiagnosed BD who presented with chronic diarrhea for 4 months, leading to the diagnosis of BD and responded well to corticosteroid therapy.

## Graphical abstract

Pathology slides showing inflammatory changes.

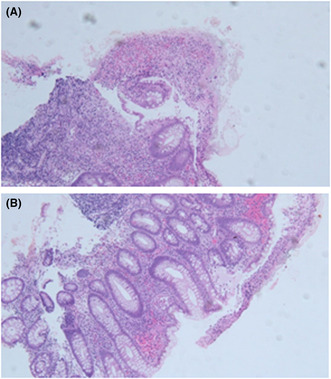

## 1 INTRODUCTION

Behçet's disease (BD) or syndrome is a chronic, recurrent, multisystem, inflammatory vasculitis disorder of unknown etiology, associated with presence of human leukocyte antigen (HLA) especially HLA‐B5. The wide clinic spectrum includes recurrent aphthous oral ulcers (97.5%), genital ulcers (65.7%), skin lesions (64.6%), ocular inflammation or uveitis as well as neurological, cardiovascular and gastrointestinal and articular involvement.^1^ It affected any age with the highest prevalence at the 30s and no gender preference. It is a worldwide disease with strong predilection for certain areas in particular the Far East, Middle East and Mediterranean basin countries. The prevalence in the United State is about 0.12–0.33 per 100,000.^2^

Gastrointestinal (GI) manifestations of Behçet's disease are of particular importance as they are associated with significant morbidity and mortality. GI manifestations usually occur 4.5–6 years after the onset of oral ulcers. The most common symptoms include abdominal pain, nausea, vomiting, diarrhea and gastrointestinal bleeding. Although oral and ileocecal involvement are most commonly described, BD may involve any segment of the alimentary tract and the various GI organs^3^ which can be challenging to differentiate from other GI disorders especially inflammatory bowel diseases. Here, we present a case of Behçet's disease who presented with GI involvement.

## 2 CASE PRESENTATION

**FIGURE 1:**
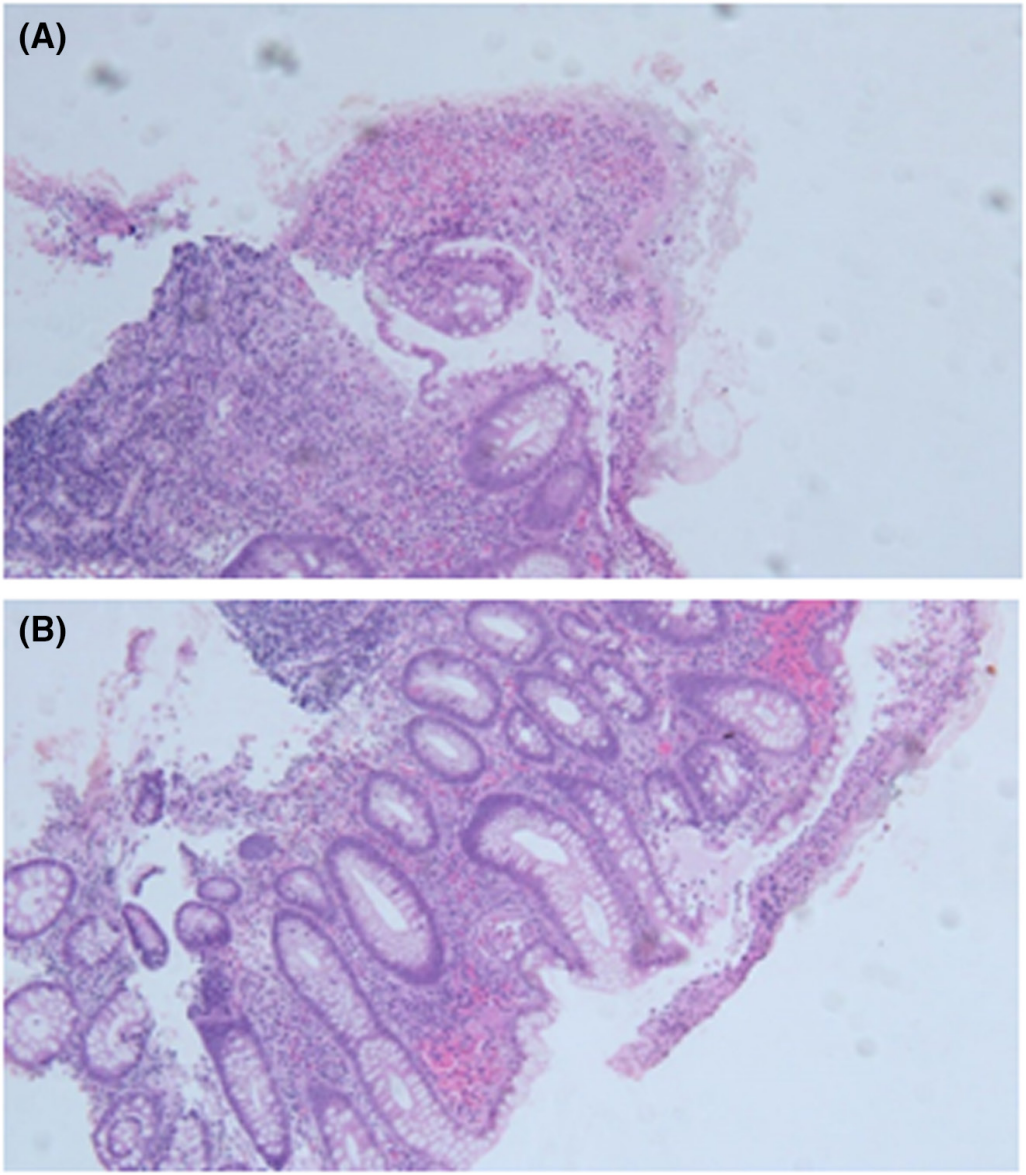
Microscopic picture of biopsies from the ulcerated ileocecal lesion showing severe acute inflammation with ulceration (A) and crypt abscesses(B)

A 32‐year‐old male patient of Mediterranean origin presented with chronic diarrhea that has been present for 4 months. He described it as watery, occasionally bloody, that occurs 8–10 times a day associated with mucous. He reported 16 kg weight loss in 4 months, recurrent oral ulcers for a year, recurrent joint pains, eye symptoms; he described right eye pain, redness, blurry vision, and light sensitivity, and sometimes a skin rash. At the site of needle pricking for blood labs, he started to develop papule around it after 24 h. Initial lab work showed elevated ESR at 55 mm/h and CRP at 10 mg/dL. CT abdomen showed thickening of the ileum and cecum. Given the alarming symptoms of chronic bloody diarrhea and weight loss, a colonoscopy was done to rule out inflammatory bowel disease; however, the biopsy showed focal ulceration (Figure 1A), cryptitis, and crypt abscesses (Figure 1B) that was atypical for inflammatory bowel disease. He was examined by an ophthalmologist, who documented evidence of uveitis in his right eye. The Rheumatology team was consulted and did a pathergy; it was positive after 48 h. The patient met the criteria for the diagnosis of Behçet's disease. The presence of recurrent large oral aphthae 1 year before presentation, evidence of uveitis, skin rash, and positive pathergy test confirmed the diagnosis. After the diagnosis, he was started on prednisone 1 mg/kg/day along with azathioprine. At the 3‐month follow‐up, the patient reported improvement of his oral ulcers, skin rash and visual symptoms. He also reported resolution of his diarrhea.

## 3 DISCUSSION

The diagnosis of Behçet's disease is challenging. No pathognomonic test can be used to make a definite diagnosis. The presence of recurrent oral aphthae at least three times in a year plus two of the following: recurrent genital aphthae, uveitis, skin lesions (pseudo folliculitis, papulopustular lesions, and erythema nodosum), and positive pathergy test defined by a papule of 2 mm or more developing after oblique insertion of a 20‐gauge needle 5 mm into the skin of the forearm after 24–48 h.^4^

Ileocecal region ulceration represents the most affected GI site followed by duodenum, jejunum, colon but rarely rectum. Two forms of intestinal involvement has been distinguished: neutrophilic phlebitis that cause mucosal inflammation and ulcer formation and large vessel disease that results in intestinal ischemia or infarction.^3^ The usual complaints of the patients include bloating, cramping, abdominal pain, diarrhea, melena, and hematochezia while weight loss and indigestion are infrequent.^2^ These symptoms can occur with other common GI diseases such as diverticular disease, inflammatory bowel disease or colorectal cancer which should be interpreted carefully.

Differential diagnosis of intestinal lesions in this case is inflammatory bowel disease particularly Crohn's disease. These two diseases share many similarities including clinical, pathological, endoscopic, and radiological findings. Both these diseases commonly have a young age of onset, nonspecific gastrointestinal manifestations, similar extraintestinal manifestations include oral ulcer, erythema nodosum, arthritis and uveitis and a chronic, waxing and waning course.^5^ Crohn's disease patients tended to have multiple‐site involvement, whereas lesions of intestinal BD were more likely to be confined to the ileocecal region. Moreover, the morphology of the lesions was different from each other. Ulcers of intestinal BD were always round or oval in shape >2 cm in size, focal distribution and usually less than five ulcers. On the contrary, ulcers of Crohn's disease were mostly irregular, longitudinal ulcers with cobblestone appearance, segmental or diffuse involvement. Given transmural inflammation resulting in fistula, stricture of bowel, abscess and anorectal involvement, this could be more distinct in Crohn's disease patients compared with intestinal BD.^6^ In term of histopathologic findings, BD showed non‐specific neutrophilic or lymphocytic phlebitis while focal cryptitis and epithelioid granuloma are found in Crohn's disease.^3^

The goal of management of BD is to tailor medical therapy to the level of clinical severity to achieve and maintain remission and prevent surgical intervention which the scoring system known as Disease Activity Index of Behcet's Disease^7^ are used. Treatment for intestinal BD starts with 5‐ASA for mild to moderate disease. However, if fails, corticosteroids should be used, gradually tapered and discontinued as there are steroid‐sparing agents to be used including TNF inhibitors for moderate to severe disease. In case aforementioned therapies fail, concomitant immunomodulators should be considered.^8^

## AUTHOR CONTRIBUTIONS

**Marawan Elmassry:** Investigation; methodology; resources; writing – original draft. **Sayed Matar:** Investigation. **Jerapas Thongpiya:** Methodology; writing – original draft. **Pitchaporn Yingchoncharoen:** Writing – original draft; writing – review and editing. **Mostafa Abohelwa:** Writing – review and editing. **Sameer Islam:** Writing – review and editing.

## FUNDING INFORMATION

This research received no specific grant from any funding agency in the public, commercial, or not‐for‐profit sectors.

## CONFLICT OF INTEREST STATEMENT

The authors have no financial conflicts to disclose.

## CONSENT

Written informed consent was obtained from the patient to publish this report in accordance with the journal's patient consent policy.

## Data Availability

The authors confirm that the data supporting the findings of the case study are available within the article. Raw data that support the findings of this case study are available from the corresponding author, upon reasonable request.
